# Optimizing the L/S Ratio in Geopolymers for the Production of Large-Size Elements with 3D Printing Technology

**DOI:** 10.3390/ma15093362

**Published:** 2022-05-07

**Authors:** Joanna Marczyk, Celina Ziejewska, Kinga Pławecka, Agnieszka Bąk, Michał Łach, Kinga Korniejenko, Izabela Hager, Janusz Mikuła, Wei-Ting Lin, Marek Hebda

**Affiliations:** 1 Faculty of Material Engineering and Physics, Cracow University of Technology, Jana Pawła II 37, 31-864 Cracow, Poland; joanna.marczyk@pk.edu.pl (J.M.); celina.ziejewska@pk.edu.pl (C.Z.); kinga.plawecka@pk.edu.pl (K.P.); agnieszka.bak@pk.edu.pl (A.B.); michal.lach@pk.edu.pl (M.Ł.); janusz.mikula@pk.edu.pl (J.M.); mhebda@pk.edu.pl (M.H.); 2 Faculty of Civil Engineering, Cracow University of Technology, 31-155 Cracow, Poland; ihager@pk.edu.pl; 3 Department of Civil Engineering, National Ilan University, No. 1, Sec. 1, Shennong Road, Yilan City 260, Taiwan; wtlin@niu.edu.tw

**Keywords:** geopolymer, frost resistance, liquid/solid ratio, additive manufacturing, 3D printing

## Abstract

Geopolymer concretes can be a viable alternative to conventional Portland cement-based materials. In their design, it is important to maintain an appropriate liquid-to-solid ratio (L/S), which affects several properties, such as the compressive strength, water absorption, and frost resistance. The objective of this paper is to analyze the influence of the fly-ash and metakaolin precursor types for three different L/S ratios: 0.30, 0.35, and 0.45. The results of the physical and mechanical properties, including the apparent density and compressive strength, as well the durability parameters, including frost resistance and water penetration depth, are presented in this paper. It was found that as the L/S ratio decreased, the average compressive strength increased for all materials. After freeze–thaw cycles, decreases in the compressive strength properties were observed for all types of materials—metakaolin- and fly ash-based—irrespective of the L/S ratio. Moreover, the frost resistance of geopolymers increased with the increase in the L/S ratio. The printability of the mixes was also verified in order to confirm the application of the developed materials to additive manufacturing processes.

## 1. Introduction

Geopolymer materials are extremely interesting materials that can replace traditional Portland cement-based concretes [1]. Although they have been known for at least several decades, researchers are still conducting research into improving the optimization of their manufacturing parameters and the selection of parameters depending on the raw material used in their production [2,3,4,5,6,7,8]. A very important driver for the implementation of geopolymer concretes is the perspective of substantially reduced CO_2_ emissions in their production process compared to the CO_2_ emissions in the production of Portland cement. However, continuous research is needed to develop a universal testing methodology and to optimize the chemical composition of activators and the L/S ratio for a particular type of raw material (often raw materials found only locally are used) [9].

In fact, the geopolymer materials themselves can be synthesized at increased or ambient temperatures by alkaline activation using industrial waste (fly ash [10], slag [11,12]) or materials of geological origin (metakaolin [13], volcanic tuff [14]). Globally, access to base materials (raw materials) is very common and attractive, due to their wide use in industry. As a result, demand for base materials is on an upward trend [15,16]. The main factors influencing the geopolymerization process are the type of base material, size, particle size, and the activator used [4,17,18,19,20]. Kastiukas et al. observed that differences in Na_2_SiO_3_ content affect the workability, curing time, and compressive strength of the produced geopolymer [21].

Sodium hydroxide (NaOH) and sodium silicate (Na_2_SiO_3_, water glass) are most commonly used as alkaline activators [22]. Studies have shown that the performance of a geopolymer binder depends on several parameters, including the activator concentration, activator ratio, or liquid-to-solid ratio, among others [23,24,25,26]. One of the key parameters affecting a number of properties and their subsequent use is the liquid-to-solid ratio [27,28,29]. However, in the case of geopolymers, the L/S ratio can be said to be of more significance. Geopolymers are made from different types of, often non-normative, raw materials that vary in hydrophobicity, porosity and moisture content [30,31].

Some work indicates that geopolymer materials exhibit good compressive strength at an alkali activator factor of 2.5 [22,32]. Kwek et al. concluded from their results that the optimum parameters for producing the above geopolymer were a liquid-to-solid ratio of 0.6 and an alkaline activator ratio of 2.5 [33]. Wand et al. observed an increase in the strength properties of the geopolymer as the L/S ratio increased. However, a geopolymer with a Si/Al ratio higher than 3 indicated worse chemical stability in air than a geopolymer with a Si/Al ratio lower than 2.5 [34]. Wan et al., investigating the microstructure of metakaolin-based geopolymers and geopolymerization reactions at various Si/Al ratios, observed that soluble silicates promoted the dissolution of metakaolin at Si/Al ratios of less than 2:1.2 [35].

Currently, research is being conducted into the effects of low temperatures on the properties of geopolymers. [36,37]. Fu et al., in their study, described the influence of freezing cycles on the mechanical properties of alkali-activated slag concrete (ASC). The results indicated that ASC exhibited great resistance to freezing and thawing, as revealed by high compressive strength results (approximately 90 MPa) [38]. Another study investigated the frost resistance of a geopolymer based on class F slag and fly ash reinforced with PP, PVA, and steel fibers. The addition of PVA fibers at 0.3% by volume had the best effect on improving the frost resistance and mechanical properties [39]. Nazarpour and Jamali conducted a study on the application of recycled aggregates in geopolymer concrete as a replacement for coarse aggregate. The results of the freeze–thaw cycle revealed that there was no significant effect on the compressive strength of the geopolymer concrete [40]. In the study, Degirments tested the resistance of pozzolana-based geopolymer mortars to fire and freeze–thaw cycles. The average compressive strength values of all samples were lower compared to the values obtained for the reference samples. The exceptions were samples containing ground granulated blast furnace slag [41].

Recently, 3D printing has become increasingly popular, especially in the construction industry [42,43]. In order to ensure an optimal printing process of a geopolymer compound, it is important to select the appropriate base materials [44]. Relevant factors when designing a mixture for printing include not only the workability of the mass but also the curing process of the printed component. Therefore, a mixture designed for 3D printing should have a low viscosity when flowing through the extruder nozzle and a high yield stress immediately after printing so that the manufactured part does not disintegrate [45,46].

Geopolymer concretes can be a practical alternative to conventional Portland cement-based concretes. When designing them, it is important to maintain an appropriate L/S ratio, which affects a series of properties, such as frost resistance, compressive strength, and water absorption. This study focuses on the effects of different L/S ratios in fly ash-, metakaolin-, sand, and fine basalt aggregate-based geopolymer materials on compressive strength, frost resistance, and water penetration depth.

## 2. Materials and Methods

### 2.1. Materials

Class F fly ash (Skawina Combined Heat and Power Plant, Skawina, Poland) and metakaolin KM 60 (Keramost, Kadaň, Czech Republic) were used as precursors. Table 1 describes the chemical composition of fly ash and metakaolin. The raw materials were mixed with quartz sand (Świętochłowice, Poland) with the following chemical composition: 90.0–90.3% SiO_2_, 0.4–0.7% Al_2_O_3_, max. 0.2% Fe_2_O_3_, 0.17% CaO, 0.08–0.1% TiO_2_, 0.01% MgO. Fine basalt aggregate (PGP “BAZALT” S.A., Wilków, Poland) with a size of 2–5 mm was used as the reinforcement material. The basalt aggregate had the following chemical composition: 44–52% SiO_2_, 12–15% Al_2_O_3_, 10–16% CaO, 5–15% FeO, 5–12% MgO. A comprehensive characterization of the raw materials was conducted and described in a previous paper [47].

### 2.2. Preparation of Specimens

The raw materials, i.e., fly ash (FA) or metakaolin (MK), were mixed with quartz sand at a ratio of 1:1. This is not only an economical solution but also contributes to higher strength. The prepared mix was activated with an activator solution, which consisted of 10-molarsodium hydroxide (NaOH) solution and an aqueous solution of sodium silicate (R-145). The ratio of the sodium base solution to the water glass solution was 1:2.5. All constituents were mixed in a GEOLAB cement mortar mixing machine (GEOLAB, Warsaw, Poland) for about 15 min until a uniform paste was obtained. Mixes of dry, plastic, and liquid consistencies, depending on the liquid/solid ratio, were prepared. Mixes of plastic consistency containing basalt aggregate were also produced. The aggregate was added at the end of the mixing of the paste. The mix contained FA/MK, sand, and aggregate in the proportions of 40:30:30 (wt.%). The designed geopolymer mixes are presented in Table 2.

The prepared geopolymer masses were cast into molds. For removing air bubbles, the molds with the material were placed on a vibrating table. The samples were cured at 75 °C for 24 h, then cooled to room temperature, removed from the molds, and stored under ambient conditions.

### 2.3. Methods

A PANalytical Aeris (Malvern PANalytical, Lelyweg 1, Almelo, The Netherlands) instrument was applied to explore the mineralogical compositions of the produced samples. The quantitative analysis was conducted by means of the Rietveld method, which was implemented in the HighScore Plus software (Version: 4.8, Malvern PANalytical B.V., Almelo, The Netherlands). The Rietveld method uses the least squares method. It is performed in order to improve the theoretical line profile so that it fits the measured profile [48]. The International Center for Diffraction Data (ICDD) PDF–4+database was used during the analysis. Measurements were recorded in the range of 10–80° with a step size of 0.003° (2θ) and time per step of 340 s, using Cu Kα radiation.

A batch of samples of 5 cm × 5 cm × 5 cm was used to execute the compressive strength tests in accordance with the PN-EN 12390-3:2019 standard, after 7 days of curing.

The geometrical density was computed before mechanical tests by dividing the mass of the specimen by its volume.

The mass change was registered using the Radwag XA 60/220/Y balance (RADWAG Wagi Elektroniczne, Radom, Poland).

The freeze–thaw resistance tests were carried out in accordance with the PN-B-06265 standard. Firstly, six geopolymer specimens with the dimensions of 50 mm × 50 mm × 50 mm for each mix were made. Then, all samples were immersed in water for seven days under ambient conditions. After that, some of the samples were placed in a freezer at a temperature of −18 ± 2 °C for 12 cycles of freeze–thaw. Each cycle consisted of freezing for a minimum of 4 h and thawing the samples in water for 2–4 h. The compressive strength, mass loss, and visual appearance of all the samples were determined afterwards [49,50].

Lastly, the water permeability of the geopolymer specimens was carried out in accordance with PN-EN 12390-8, using six 150 × 150 × 150 mm cubic samples per blend.

## 3. Results and Discussion

### 3.1. X-ray Diffraction

**Figure 1:**
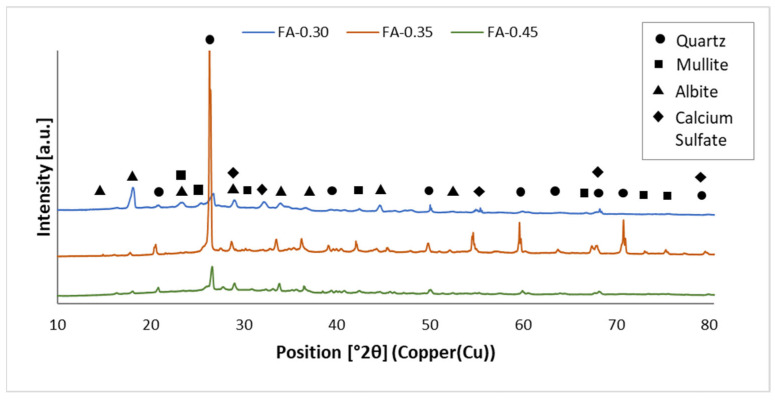
Phases present in fly ash-based geopolymers.

**Figure 2:**
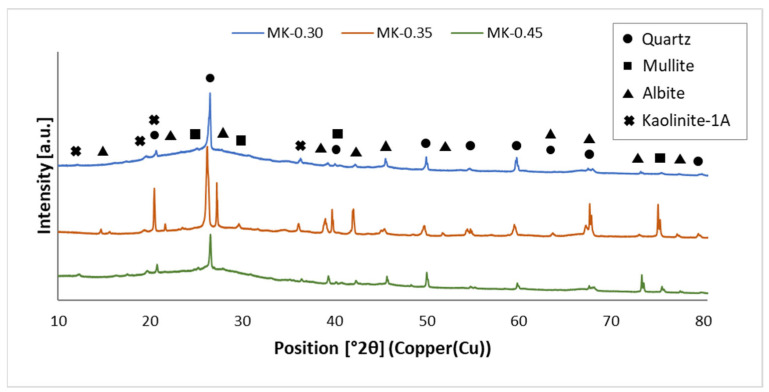
Phases in a metakaolin-based geopolymer.

The results of the qualitative and quantitative X-ray analyses performed for the fly ash- and metakaolin-based geopolymers are shown in Figure 1 and Figure 2 and Table 3. The XRD spectra obtained for both types of geopolymer show the attendance of phases especially rich in Al and Si, such as quartz (SiO_2_, ref. code: 01-070-2517), mullite (Al_6_Si_2_O_13_, ref. code: 00-015-0776) and albite (NaAlSi_3_O_8_, ref. code: 00-020-0572). Diffraction peaks derived from calcium sulfate (CaSO_4_, ref. code: 00-055-0953) were identified in the fly ash-based geopolymer samples, while kaolinite-1A (Al_2_Si_2_O_5_(OH)_4_, ref. code: 00-058-2004) was detected for the metakaolin-based geopolymers.

Figure 1 shows the X-ray diffraction spectra of fly ash-based geopolymers. A sharp peak recorded at 2θ = 26° indicates the attendance of crystalline phases in the geopolymer. As a result of aluminosilicate compounds, the crystalline quartz phase refines the physical and mechanical properties. Stable phases that do not dissolve easily in alkaline solutions are quartz and mullite. This results in both a reduction in the reactivity of ashes and the level of geopolymerization [51]. In the XRD patterns, there is a wide peak between 20° and 40° 2θ, indicating that a geopolymerization reaction occurred between the alkali activator and fly ash. As a result, a gel with amorphous characteristics was formed [52,53]. A similar phenomenon was identified by Lu et al. [54] and Chen et al. [52]. The polymerization of aluminosilicate and an alkaline activator under high-temperature curing conditions produces Albite [53]. XRD patterns of fly ash-based geopolymers showed a high presence of calcium sulfate. The combustion of calcite contained in coal produces calcium sulfate in the form of anhydrite, which occurs in fly ash The formation of this mineral is due to the high content of CaO and SO_3_ in the fly ash. Research shows that concrete comprising anhydrite-abundant fly ash demonstrates higher strength than concrete made from anhydrite-free fly ash [55].

XRD patterns of metakaolin-based geopolymers are shown in Figure 2. All three samples show similarly scattered humps between 20° and 30° 2θ, enabling an amorphous structure. A similar phenomenon was observed by Wang et al. [56]. This diffuse peak can be attributed to the attendance of amorphous aluminosilicate gel, which is the major combined phase occurring in geopolymers [57]. In addition, unreacted amorphous MK particles may contribute to a rise in the amorphous structure of geopolymers [56,58]. The XRD patterns of geopolymers indicate the attendance of distinctive peaks, such as quartz. This indicates that the crystalline phases are not solved in the alkaline solution. They are present in the geopolymer binder in the form of inactive loads and do not participate in the geopolymerization reaction. Only amorphous phases in the starting materials are reactive and involved in geopolymerization reactions [57]. Although quantitative XRD analysis is not as accurate, it can provide the necessary information. The results confirm that stable albite crystals are generated in geopolymer samples.

Multiple peaks were observed. For fly ash-based geopolymers, mullite and albite were detected at 24° 2θ, albite and calcium sulfate near 29° 2θ, and quartz and calcium sulfate at 69° and 80° 2θ. For metakaolin-based geopolymers, the presence of phases such as quartz and kaolinite at the same angular position close to 22° 2θ was also visible in the research by Burduhos Nergis et al. [59]. Quartz and mullite were detected at 40° 2θ, as well as quartz and albite at angles of 64° and 69° 2θ.

Comparing the XRD spectra shown in Figure 1 and Figure 2 with the XRD spectra of the raw materials [47], it can be concluded that the peaks from quartz, mullite, kaolinite, and calcium sulfate appeared at the same angular positions. A characteristic asymmetric hump was observed both in the XRD pattern of the MK raw material and in the metakaolin-based geopolymers in the angular range of 20–30° 2θ, which indicates the amorphous phase related to aluminosilicate glass. After the geopolymerization process, the presence of the albite phase was detected in the geopolymer samples. Compared to the XRD patterns of the raw materials, higher-intensity albite peaks were detected in the geopolymers at the angular positions of 22° and 27° 2θ. Albite in the geopolymers was formed as a result of the reaction of an aluminosilicate compound with an alkaline solution during the thermal curing.

### 3.2. Densities of Geopolymer Samples

**Figure 3:**
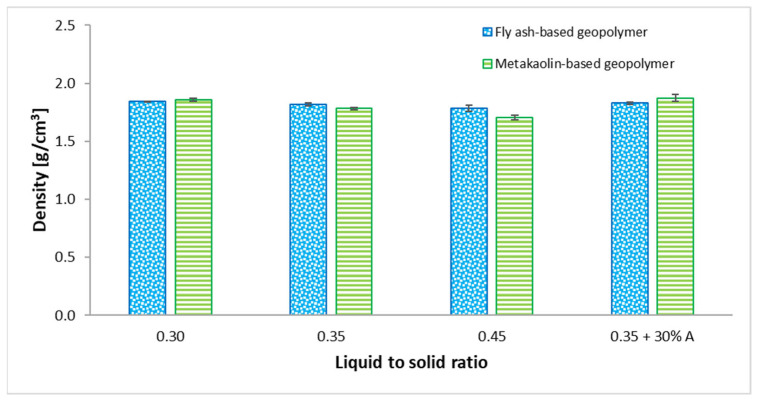
Effects of used mix proportions on densities of geopolymers based on fly ash and metakaolin.

The densities of the fly ash-based and metakaolin-based geopolymer samples are shown in Figure 3.

The geometrical densities of geopolymers based on fly ash using 0.30, 0.35, and 0.45 liquid-to-solid ratios were 1.84, 1.82, and 1.79 g cm^−3^, respectively. In the same order for metakaolin-based samples, 1.86, 1.78, and 1.71 g cm^−3^ were the density values. The geometrical density ranged from 1.71 g cm^−3^ (for MK–0.45) to 1.87 g cm^−3^ (in the case of MK–0.35 + 30% A). The lowest density was achieved using an L/S ratio equal to 0.45 for both matrix material metakaolin and fly ash. It was found that the liquid-to-solid ratio affects the density of obtained samples. In paper [33], researchers studied the effect of increasing the liquid-to-solid ratio from 0.5 to 0.85 in geopolymer samples. They showed that densities of the geopolymers decreased with increases in the liquid-to-solid ratios. This phenomenon is caused by the condensation process, which takes place in the polymerization reaction. The low content of the alkaline activator results in a delayed geopolymer reaction, reducing the hydration process. Similarly, Shi Ying et al. observed that the densities of geopolymers decreased as the L/S ratio increased [33]. However, the densities of samples with the addition of basalt aggregate were 0.83 g cm^−3^ and 0.87 g cm^−3^ for fly ash-based samples and metakaolin-based samples, respectively. There is no significant difference in the obtained results between the samples with metakaolin and fly ash.

### 3.3. The Compressive Strength of Geopolymers

**Figure 4:**
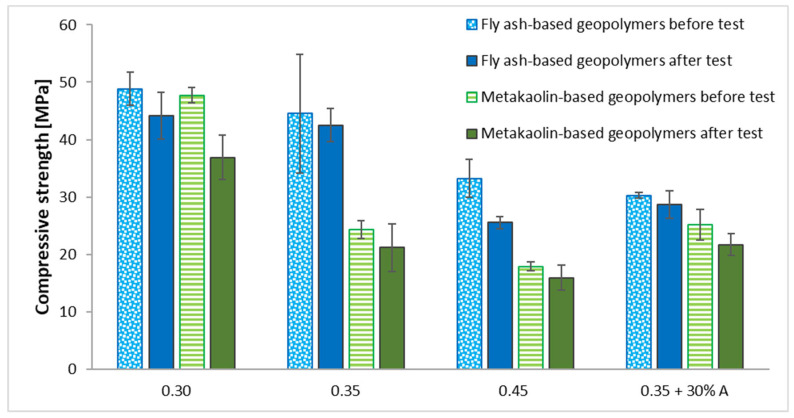
Dependence of compressive strength between samples before and after freeze–thaw resistance test of fly ash- and metakaolin-based geopolymers.

The compressive strength of geopolymers before and after 12 cycles of freeze–thaw were compared in Figure 4. The strength values of all fly ash-based samples were higher as compared to the metakaolin-based geopolymers produced using the same L/S ratio. For each of the samples, the compressive strength was greater before the freeze–thaw resistance test than after it. However, the obtained results explicitly show that the higher L/S ratios in the geopolymer samples resulted in a decrease in the compressive strength. Similar relationships were presented by other authors in their works [25,60,61]. Aman et al. found that the compressive strength of geopolymer samples increased until the optimum liquid-to-solid ratio was reached [62]. The geopolymer samples with 0.3 L/S ratios achieved the highest compressive strength independently of the used base material. Adding basalt aggregate at a ratio of 30% provided a decrease in the compressive strength in the case of samples with fly ash. However, the opposite trend was observed when the basalt aggregate was applied to the metakaolin-based geopolymer. In these types of samples (MK–0.35 + 30% A), adding the basalt aggregate resulted in little increase in their compressive strength as compared to the samples manufactured with the same L/S ratio (MK–0.35). Sahin et al. [63] examined the effect of adding, among others, basalt sand into the metakaolin-based geopolymers. They showed that the addition of basalt sand improved the mechanical properties of samples. Moreover, Sahin et al., in their other work [64], wrote that basalt aggregate is an economical product due to its low price and large amount, in addition to providing high strength to metakaolin-based geopolymers. Thus, the addition of basalt aggregate led to an increase in the compressive strength of geopolymers with a metakaolin base.

The average compressive strength reductions after the freeze–thaw resistance test of the fly ash-based geopolymers amounted to 9.5, 4.5, 23.1, and 5.2 % for FA–0.30, FA–0.35, FA–0.45, and FA–0.35 + 30% A, respectively. Moreover, in the case of the metakaolin-based samples, the average compressive strength reductions after 12 freeze–thaw cycles reached the values of 22.8, 12.7, 11.0, and 13.6 % for MK–0.30, MK–0.35, MK–0.45, and MK–0.35 + 30% A, respectively.

**Figure 5:**
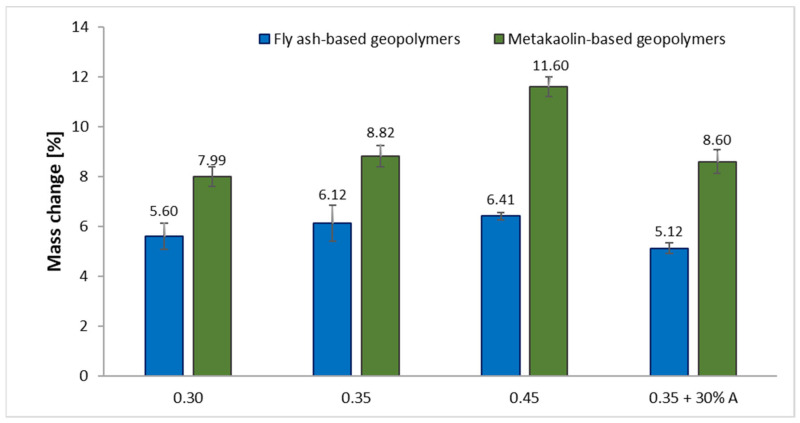
Effect of applied freeze–thaw cycles on the mass changes in geopolymer samples.

The mass changes in the geopolymer samples based on both fly ash and metakaolin after 12 freeze–thaw cycles are shown in Figure 5. The values represent the gains in weight.

The percentages of mass changes were computed to analyze the effects of using freeze–thaw cycles on the geopolymer samples. The obtained results verified that the masses of all the samples increased after the tests, which means that the samples gained weight. It was noticed that the masses of specimens increased as the liquid-to-solid ratios in the geopolymer samples increased. Moreover, the mass changes in the metakaolin-based samples were greater in comparison to the corresponding fly ash-based geopolymers. This was due to the fact that the samples absorbed water inside the microcracks as well as the pores [65]. The present study consisted of 12 freeze–thaw cycles. Ping et al. [66] showed that during the first forty freeze–thaw cycles, the masses of the samples significantly increased as a result of expanding cracks and spreading new cracks. Similarly, Bumanis et al. [67] assessed the effectiveness of freeze–thaw testing methods by investigating high-strength concrete with the addition of cementitious materials. They showed that the masses of the investigated samples continuously increased up to the 12th freeze–thaw cycle. In the next cycles, the masses still increased, but the increases were smaller.

On the other hand, the addition of basalt aggregate provided the samples with lower value increments as compared to the specimens with the same L/S ratio without additives. The relationship between the mass changes and compressive strength of the geopolymer samples after the freeze–thaw resistance test was observed. The decrease in the compressive strength of the geopolymers was associated with the proportional increase in the sample mass.

### 3.4. Evaluation of the Morphology of Samples after Frost Resistance Tests

**Figure 6:**
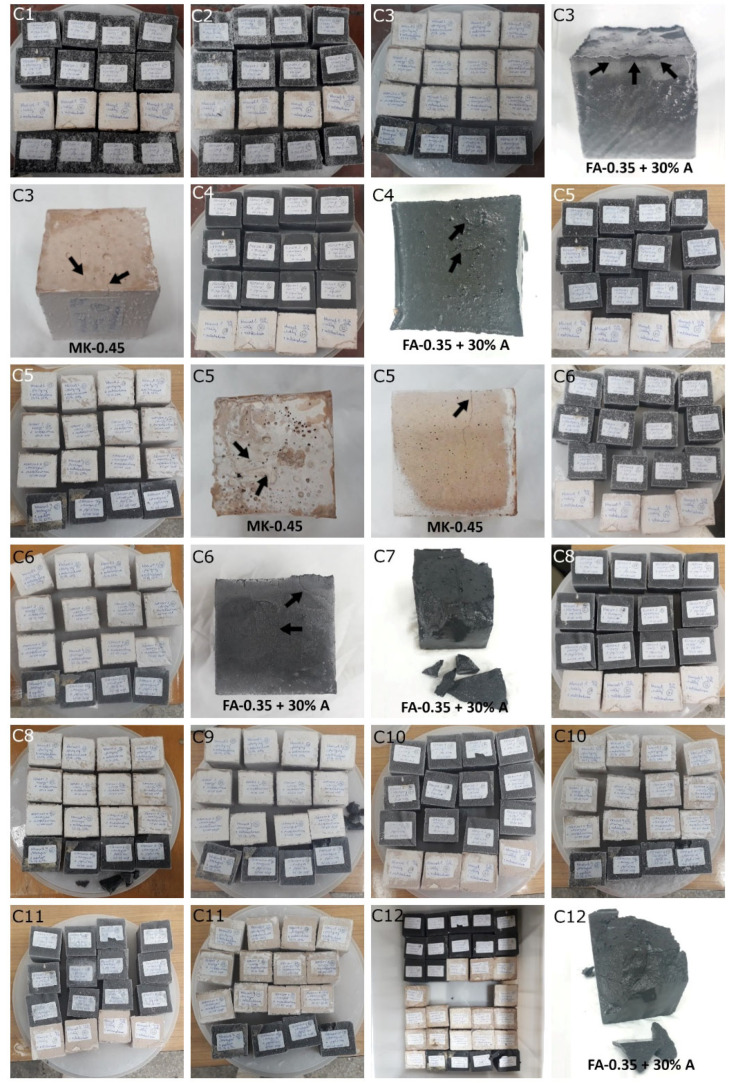
Damaged morphology of geopolymers in successive freeze–thaw cycles. C1–C12 indicate successive freeze-thaw cycles.

**Figure 7:**
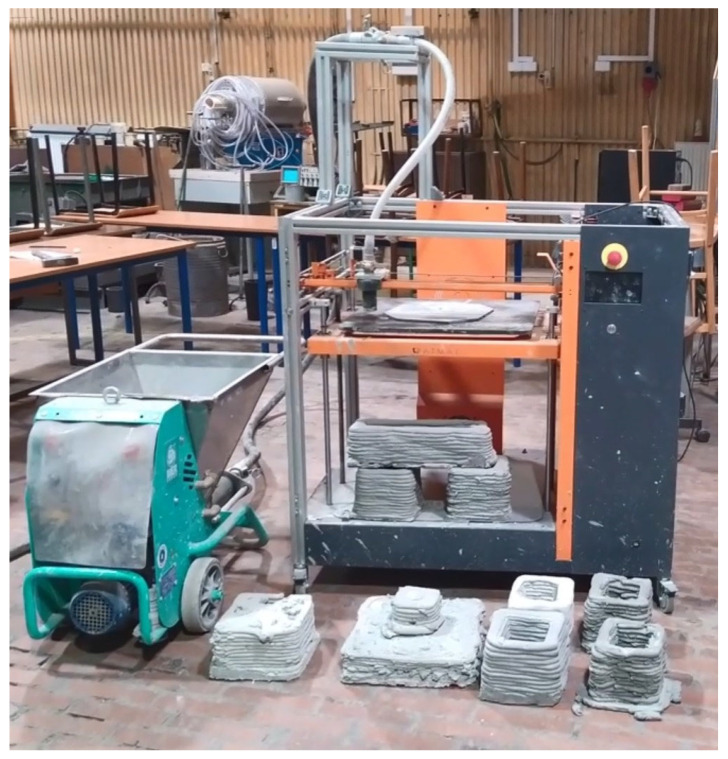
ATMAT Galaxy 3D printer.

Figure 6 shows the failure morphology of samples after consecutive freeze–thaw cycles. The marking of C along with the number corresponds to the cycle after which the visual assessment was performed. In Figure 7, the overall appearances of the samples are presented, which allows for observing the progress of degradation. Moreover, some representative samples were selected to show a more detailed progression of the cracking (cycles C3, C5, C6, C7, and C12). 

There are two common freeze–thaw malfunction modes: surface peeling and inner cracking [68]. The first noticeable cracks appeared after three freeze–thaw cycles in the geopolymer sample with the addition of basalt aggregate (FA–0.35 + 30% A) and the MK–0.45 sample. After four cycles, further cracks and surface peeling appeared in the FA–0.35 + 30% A sample. After five freeze–thaw cycles, a slender and long crack was observed on the side wall of the MK–0.45 sample. Then, changes were noted for the FA–0.35 + 30% A geopolymer, for which subsequent cracks appeared after six cycles. Moreover, after seven freeze–thaw cycles, the geopolymer crumbled, which showed strong surface exfoliation. The next major damage to the fly ash-based geopolymer with aggregate was observed after all 12 cycles. Generally, after running 12 freeze–thaw cycles, the MK–0.45 specimens showed cracks on the edges and sides. Comparable results were acquired by Yuan et al. [39] for samples with 15 freeze–thaw cycles. However, the FA–0.35 + 30% A sample was completely damaged, which may mean that it had a reduced frost resistance. These results are integrated with the compressive strength results. In other geopolymer samples after 12 freeze–thaw cycles, no spalling or surface cracks were observed. Török et al. [68] carried out frost resistance tests for mortars and porous limestone. For the mortar samples, they noticed damage after 10 cycles. However, severe damage appeared after 25 cycles. Similarly, Temuujin et al. [69], in their work, showed that the tested geopolymer concretes, activated with 50% sodium silicate solution and 50% NaOH, were characterized by low frost resistance. The samples cracked after five freeze–thaw cycles. In general, the densification of the samples can limit the ingress of water into their microstructures. As a result, the microstructural damage is reduced. Such damage may be due to augmented inner hydraulic stress caused by the enlargement of the pore ice volume [70]. 

Table 4 shows representative samples tested for compressive strength before and after 12 freeze–thaw cycles.

The use of 12 freeze–thaw cycles during frost resistance testing resulted in decreases in the compressive strength of the geopolymer specimens. During the tests, crack propagation and spalling appeared in some samples. As a result, specimens that were compressed after 12 cycles cracked and failed more easily.

### 3.5. Water Permeability

The water permeability test effects obtained for the FA–0.30 and MK–0.30 mixes are indicated in Table 5. The geopolymer mixes with the highest compressive strength were selected for this study.

A reduction in the profundity of water penetrance was observed with the change in the raw material from metakaolin to fly ash. For the FA–0.30 samples, the depth of water penetration was 14 mm. The geopolymers were not permeated after 24, 48, and 76 h of testing. The MK–0.30 samples were also not soaked after 24 and 48 h of testing. However, they were soaked after 76 h. Thus, the depth of water penetration for the MK–0.30 samples was 150 mm. The results obtained for the geopolymer samples based on fly ash show a significant decrease in the water penetration depth, about 90% less compared to the metakaolin-based samples. The depth of water penetration provides information about the durability of the geopolymer. Moreover, the penetration depth and the compressive strength are correlated [71]. The lower permeability of concrete is characterized by increased resistance to chemical assaults. Soluble salts with chloride ions can cause corrosion when water gets into the sample [72]. Duan et al. [73], in their work, noticed that geopolymers were characterized by lower water absorption than samples from OPC. Metakaolin-based geopolymers were characterized by slightly higher water absorption than fly ash-based geopolymers.

## 4. Large-Format 3D Printing

The results presented in this work were realized in order to determine the optimal properties of geopolymers that will ultimately be manufactured in large-format 3D printing technology. Figure 7 shows the Galaxy printer (ATMAT, Krakow, Poland) with a working area of 700 × 700 × 1000 mm and a nozzle diameter of 15 mm coupled with the SMALL-50 plastering machine (IMER Group, Italy), which were used for the 3D printing process.

**Figure 8:**
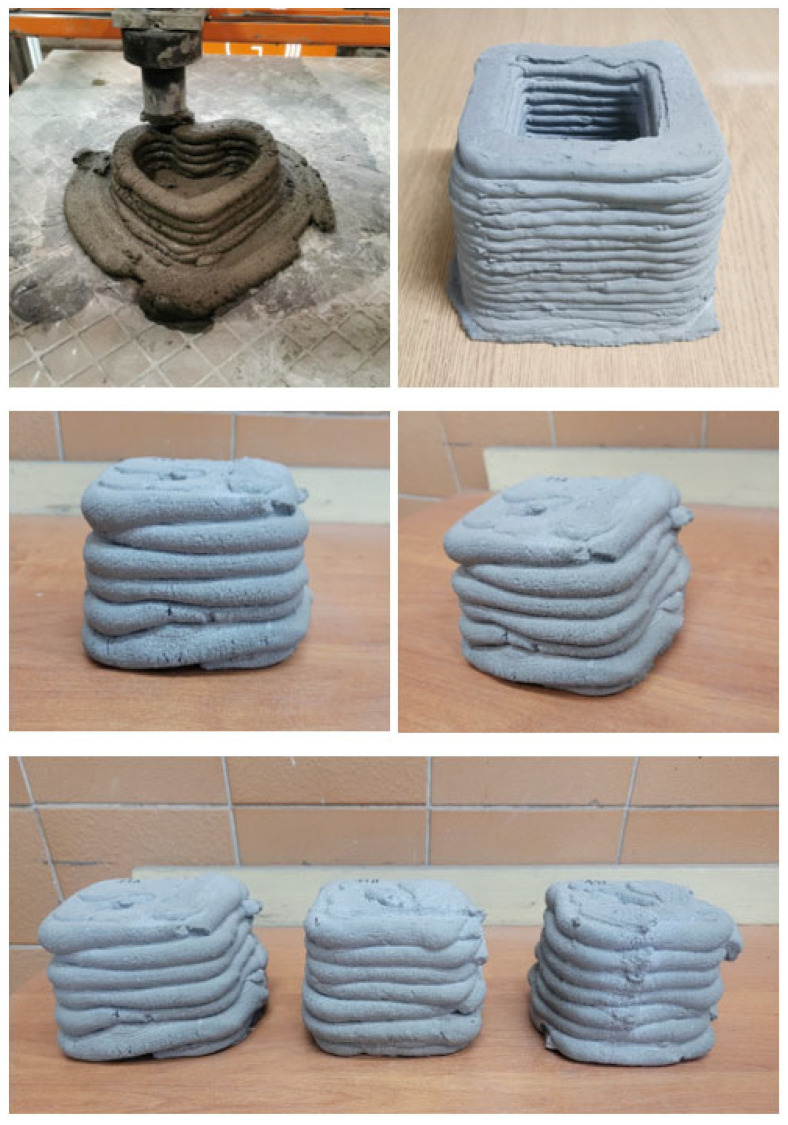
Sample printouts from a 3D printer.

Ambient temperature-cured example printouts are presented in Figure 8. Ultimately, the production is to be based on advanced large-format 3D printing on a printer with a working area of 11.0 × 5.85 × 0.5 m.

Studies were carried out in terms of the selection of the parameters and the material compositions for the production of the geopolymer mix. The appropriate L/S ratio of the geopolymer blend had an imperative impact not only on the continuity of the extrusion process but also on the stability of the printouts. The research was carried out under grant no. POIR.04.01.04-00-0096/18-00, which concerns the development of 3D technology for the production of construction and prefabricated facade elements made of concrete composites and geopolymers.

## 5. Conclusions

Issues related to determining the optimum L/S ratio are extremely important for geopolymer materials, especially in the context of their application. In the case of precast molding, the L/S ratio should provide a consistency that allows the mold to be filled accurately and maintain the appropriate strength properties. However, in the case of using geopolymers in 3D printing technology, which has recently become more and more common, it is important that the consistency should allow for maintaining a stable spatial structure until the geopolymer sets. Therefore, the L/S ratio should be smaller than for the mold casting. Despite many studies and research on this issue, L/S ratios for geopolymers should be determined each time for each raw material due to the fact that there are significant differences in even the water content of fly ash or other raw materials.

The study of geopolymer materials based on fly ash and metakaolin confirmed that the compressive strength decreases with an increase in the L/S ratio, and their frost resistance also decreases. It was observed that this relationship is more pronounced for geopolymers based on metakaolin.

The geopolymer samples with a 0.3 L/S ratio achieved the highest compressive strength independently of the used base material. Adding basalt aggregate at a ratio of 30% provided a decrease in the compressive strength in the case of samples with fly ash. However, the opposite trend was observed when the basalt aggregate was applied to the metakaolin-based geopolymer. In these types of samples (MK–0.35 + 30% A), adding the basalt aggregate resulted in little increase in their compressive strength as compared to samples manufactured with the same L/S ratio (MK–0.35).

The average compressive strength reduction after the freeze–thaw resistance test of fly ash-based geopolymers amounted to 9.5, 4.5, 23.1, and 5.2% for FA–0.30, FA–0.35, FA–0.45, and FA–0.35 + 30% A, respectively. Moreover, in the case of metakaolin-based samples, the average compressive strength reduction after 12 freeze–thaw cycles reached the values of 22.8, 12.7, 11.0, and 13.6% for MK–0.30, MK–0.35, MK–0.45, and MK–0.35 + 30% A, respectively.

This research was conducted to optimize the L/S ratio for large-format 3D printing of building materials, such as floor slabs, lintels, etc. Geopolymers are an attractive material that can be used in additive technologies only if the parameters related to the consistency of this material are properly chosen.

## Figures and Tables

**Table 1:** The chemical composition of the fly ash and metakaolin determined by X-ray fluorescence analysis, wt.% (based on [47]).

Component	Fly Ash (%)	Metakaolin (%)
SiO_2_	48.22	52.43
Al_2_O_3_	26.13	42.75
Fe_2_O_3_	7.01	1.20
CaO	5.12	0.49
K_2_O	3.48	1.30
MgO	1.72	0.18
Na_2_O	1.62	0.00
SO_3_	1.11	0.03
TiO_2_	1.11	0.31
P_2_O_5_	0.70	0.44
MnO	0.09	0.01

**Table 2:** Mix designs of geopolymer samples.

Sample		Composition				Liquid/Solid Ratio
	FA (g)	MK (g)	Sand (g)	Basalt Aggregate (g)	10-Molar NaOH/Water Glass 1:2.5 (g)	
FA–0.30	100	-	100	-	60	0.30
FA–0.35	100	-	100	-	70	0.35
FA–0.45	100	-	100	-	90	0.45
FA–0.35 + 30% A	80	-	60	60	70	0.35
MK–0.30	-	100	100	-	60	0.30
MK–0.35	-	100	100	-	70	0.35
MK–0.45	-	100	100	-	90	0.45
MK–0.35 + 30% A	-	80	60	60	70	0.35

**Table 3:** Quantitative analysis of geopolymer samples.

Phase	Quantitative Share (%)					
	FA–0.30	FA–0.35	FA–0.45	MK–0.30	MK–0.35	MK–0.45
Quartz	36.6	45.1	18.3	12.6	44.8	10.1
Mullite	18.1	28.9	20.4	12.8	8.8	9.8
Albite	21.4	4.2	20.4	59.1	41.0	67.2
Calcium Sulfate	23.9	21.8	40.8	-	-	-
Kaolinite-1A	-	-	-	15.6	5.3	12.9

**Table 4:** Photographs of samples after the compressive strength test.

Designation	Reference Sample	After the Compressive Strength Test	
		Samples Not Subjected to the Freeze–Thaw Cycles	Samples after 12 Freeze–Thaw Cycles
FA–0.30	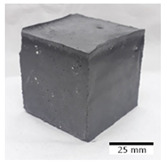	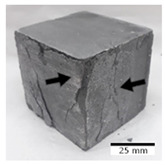	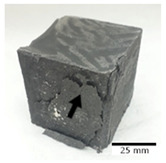
FA–0.35	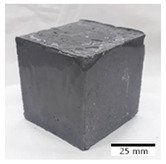	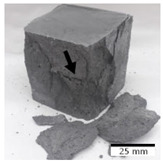	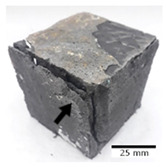
FA–0.45	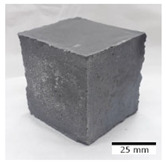	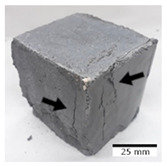	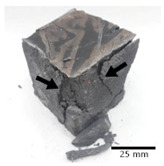
FA–0.35 + 30% A	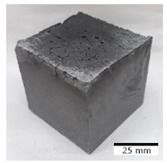	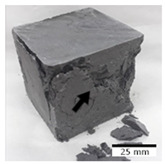	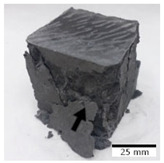
MK–0.30	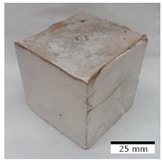	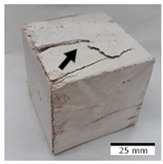	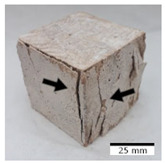
MK–0.35	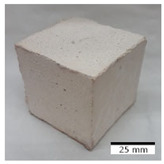	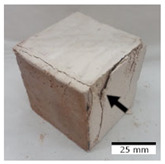	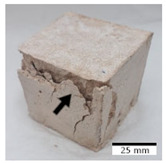
MK–0.45	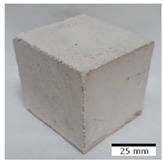	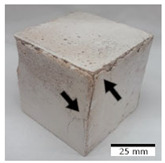	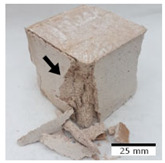
MK–0.35 + 30% A	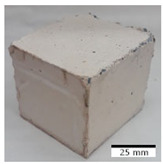	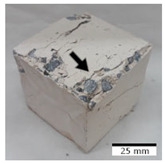	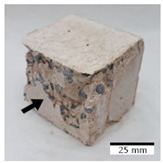

**Table 5:** Depth of water penetration of FA–0.30 and MK–0.30 geopolymers.

Sample	Time (h)			Depth of Water Penetration (mm)
	24	48	76	
FA–0.30	not soaked	not soaked	not soaked	14 ± 2
MK–0.30	not soaked	not soaked	soaked	150 ± 0

## Data Availability

Not applicable.
